# Successful parathyroidectomy improves cognition in patients with primary hyperparathyroidism: A prospective study in a tertiary medical center and comprehensive review of the literature

**DOI:** 10.3389/fendo.2022.1095189

**Published:** 2022-12-22

**Authors:** Auryan Szalat, Noa Tamir, Haggi Mazeh, J. P. Newman

**Affiliations:** ^1^ Department of Endocrinology and Metabolism, Hadassah Medical Center, Faculty of Medicine, Hebrew University of Jerusalem, Jerusalem, Israel; ^2^ Internal Medicine Ward, Osteoporosis Center, Hadassah Medical Center, Faculty of Medicine, Hebrew University of Jerusalem, Jerusalem, Israel; ^3^ Department of Surgery, Hadassah Medical Center, Faculty of Medicine, Hebrew University of Jerusalem, Jerusalem, Israel; ^4^ Department of Neurology, Hadassah Medical Center, Faculty of Medicine, Hebrew University of Jerusalem, Jerusalem, Israel

**Keywords:** parathyroid hormone, parathyroidectomy, cognitive performance, memory, primary hyperpaparathyroidism

## Abstract

**Context:**

The recent American and European guidelines on management of patients with primary hyperparathyroidism (PHPT) did not endorse neurocognitive evaluation as part of standard work-up and did not consider it as a surgery criterion.The neurocognitive deleterious effects of hyperparathyroidism and impact of parathyroidectomy on PHPT patients is yet to be elucidated.

**Objective:**

To evaluate specific neurocognitive functions in PHPT patients prior to parathyroidectomy and describe the changes during follow-up with serial evaluations.

**Design:**

A prospective case-control study including parathyroidectomy candidates evaluated at a tertiary teaching university hospital. Thorough neurocognitive evaluation was conducted before and 1- & 6-months following parathyroidectomy: Rey Auditory Verbal Learning Test (RAVLT), Rey-Osterrieth Complex Figure Test (ROCF), Trail Making Test A, Trail Making Test B, Addenbrooke’s Cognitive Examination-III (ACE), Frontal Assessment Battery (FAB), Beck Depression Inventory (BDI).

**Results:**

18 consecutive patients underwent successful parathyroidectomy. Various neurocognitive functions improved significantly after successful parathyroidectomy: long term auditory memory (RAVLT, p=0.008), short- and long-term visual memory (ROCF, p=0.006 and p=0.002 respectively), visual attention and complex concentration skills (trail making A, p<0.001) and executive abilities (trail making B, p=0.005). No change was identified in frontal-lobe abilities. Depression symptoms were absent or minimal prior to surgery and no significant change was observed after surgery.

**Conclusions:**

PHPT is associated with significant various neurocognitive dysfunctions when mindfully evaluated before surgery. Successful parathyroidectomy results in several neurocognitive aspect improvements. The data suggest that neurocognitive deterioration may be considered an added parathyroidectomy criterion when surgical decision is not straightforward.

## Introduction

Primary hyperparathyroidism (PHPT), the most frequent cause of hypercalcemia, is a common disease with 0.86% prevalence in the USA, and is more frequent in post-menopausal women (3-4/1 female-male ratio) (1), whereas in different western Europe countries the prevalence varies but a similar pattern of increased incidence specifically in postmenopausal women is observed (2) Nowadays, PHPT is usually diagnosed by laboratory routine tests in asymptomatic patients, but classic symptoms include kidney-related symptoms such as nephrolithiasis (15-20% of PHPT patients) and bone-related symptoms (<2% of PHPT patients) (1, 3). Non-classic symptoms include muscle weakness, fatigue, rheumatological, neuromuscular manifestations, and neurocognitive and psychiatric symptoms (4–6). Parathyroidectomy is the only definitive treatment for PHPT in patients who meet at least one of specific criterion (7, 8).

Based on the association between primary hyperparathyroidism and several impaired neurocognitive functions as well as quality of life, with improvement in the latter after successful parathyroidectomy (9–12), the Association of Endocrine Surgeons’ guidelines endorsed consideration of neurocognitive impairment for parathyroidectomy (7). However, neurocognitive disorders were not considered an indication for surgery by the recently-updated Fifth International Parathyroid Workshop guidelines (13). On the other hand, the recent 2021 European Expert Consensus on practical management of specific aspects of parathyroid disorders did not even mention neurocognitive impairment in PHPT patients as a relevant topic (14). This is due to controversies and questions that remain unanswered: the extent of altered neurocognitive functions in patients with hyperparathyroidism remains unclear, as well as which of them can improve after successful parathyroidectomy, and the timeline for maximal improvement achievement. Moreover, since the incidence of PHPT peaks during the sixth decade, it is often hard to isolate its effect on neurocognitive function, as some changes may be attributed to aging or comorbidities (15). Finally, the mechanisms leading to impaired neurocognitive functions are still unclear, and may be related to either hypercalcemia, decreased 25 hydroxy vitamin D (25OHD) or elevated PTH levels; all may impact brain activity (16).

The aim of this study was to evaluate the baseline neurocognitive performance of patients with PHPT and to prospectively evaluate the changes after parathyroidectomy *via* a wide-range battery of validated tests at 1- & 6-months following surgery. In addition, the study aims to determine the time period for symptom improvement and its association to serum calcium and parathyroid hormone (PTH) levels.

## Materials and methods

This prospective study was approved by the IRB committee (HMO-18-0558).

Participants in this study were PHPT patients who were candidates for parathyroidectomy and later completed the procedure. Participants completed a battery of validated neurocognitive tests and questionnaires before, and 1- & 6-months following surgery. In addition, clinical data were collected from patients’ medical records – demographics, symptoms, and lab results (including PTH and calcium levels before and after surgery).

Patients were recruited between 1.1.2018 and 31.12.2020 from both the General Surgery and Ear, Nose and Throat Departments at Hadassah-Hebrew University Medical Center in Jerusalem. All patients gave informed consent to participate in the study. All tests, before and after surgery, were conducted at patients’ homes (by the same interviewer (NT) over the study period) in order to ensure a quiet and comfortable environment as well as homogeneity. Total time for completing the battery of test was between 50 and 120 minutes, depending on the subjects’ performance and cooperation.

The battery of tests included the following tests in this order of execution:

### Rey auditory verbal learning test

examines short- and long- term verbal memory alongside learning abilities (17). It consists of a list of 15 words that are read out loud by the examiner and is recalled by the subject from memory alone. This process is repeated five times, then a different list of 15 words is read to and recalled by the subject. In the 7^th^ trial, the subject is required to recall the first list without hearing it again. A 20-minute break is held before the 8^th^ trial (recall of the first list is done), also without hearing the list again. In this time, other tests are performed. Points are given per word that is recalled correctly in each attempt, so that the score for each trial is 0-15, with 15 being the best possible score.

### Rey-Osterrieth complex figure

examines short- and long- term visual-spatial memory, attention, planning, and working memory (18). In this test, a large, complex image is required to be drawn in three stages: First, the image is copied by the subject. Second, the image is taken from the subject and he/she must draw it from memory. A 20-minute break is held before the second recall drawing of the image, without seeing the image again. In this time, other tests are performed. Points are given for each part of the image that is drawn, according to three parameters: whether elements are drawn correctly, whether they are drawn in the right location and whether they are the same size. Each component can be scored 0, 0.5, 1, or 2 points. The total scoring of the image is on a scale of 0-36, 36 being the highest score possible.

### Trail making test A and B

examines visual attention, mental flexibility, and executive functioning (19). Trail Making Test A requires drawing a line between numbers in an ascending pattern from 1 to 25 as quickly as possible. Trail Making Test B is similar but the connection should be from a number to a letter and back to a number, also in an ascending pattern, with numbers being from 1-13 and letters A-L. Score for each test is the time taken for subjects to complete them and the number of errors.

### Frontal assessment battery

examines various frontal-lobe related functions (20). It consists of six sections with six specific tasks scored 0-3 points each, with a maximum of 18 points being perfect performance.

### Addenbrooke’s cognitive examination-III

a comprehensive test which examines various neurocognitive functions including orientation, attention, memory, fluency, language, praxis and visuospatial functions (21). Each task in the test is scored with rules specific to that task, with total scoring of the ACE test being between 0 to 100 points, with higher score indicating better performance.

### Beck depression inventory

assesses depression (22). It is a questionnaire consisting of 21 questions that the subject answers alone without the interference of the examiner. Each question has four possible answers scored between 0-3 points, so the total of the BDI test is a sum of them and ranges between 0 to 63 points. Higher score indicates greater depression.

For each patient, scores were compared before and after (1 & 6 months) parathyroidectomy and therefore they served as their own control.

### Statistics

Data analysis was performed using SPSS version 25 software (SPSS, Inc, Chicago, IL). Descriptive statistics are presented using prevalence and percentage values for categorical variables, while continuous variables are presented with means and standard deviation. Non-continuous variables are presented by median and range. Generalized linear model was used to analyze the differences between each session and the others.

## Results

This study included 18 participants. Their demographic and clinical are detailed in **Table 1**. The mean age of participants was 67.9 ± 7.6 years and 14 (77.8%) were females. Mean serum calcium level prior parathyroidectomy was 11.1 ± 0.7 mg/dl (normal 8.5-10.5 mg/dl) and mean serum PTH level was 15.4 ± 7.6 pmol/l (normal 1.8-8.3 pmol/l). Intraoperative findings included 13 (72.2%) patients with a single adenoma, 1 (5.6%) patient with double adenomas, and 2 (11.1%) patients with four-gland hyperplasia. The mean weight of the glands removed was 683.7 ± 661 mg. All included patients were cured and completed two sessions of neurocognitive tests: one pre-operative and another 1-month post-operatively. Of these 18 participants, 13 (72.2%) also completed a third session 6-months post-operatively. **Table 2** presents the different tests scores, where improvement is defined by score elevation. Significant improvement in short- and long- term verbal memory alongside learning abilities were seen after curative parathyroidectomy (ACE 5, p=0.004; RAVLT 8, p=0.002). short- and long- term visual memory, attention, planning and working memory also improved significantly as the patients were able to draw the complex figure more precisely after observing it immediately and after a 20 minutes delay (ROCF 2 and ROCF 3 – p. value <0.001 for each test).

**Table 1:** Demographic and clinical characteristics.

Demographic and clinical characteristics		
Gender (%)	Male	21%
	Female	79%
Age ± SD (years)		67.9 ± 7.6
Serum Ca ± SD (mg/dl)	pre-op	11.1 ± 0.7
	2 weeks post-op	9.7 ± 0.7
Serum PTH ± SD (pmol/l)	Pre-op	15.4 ± 7.6
	2 weeks post-op	7.9 ± 5.3
Vitamin D pre-op ± SD (ng/ml)		32 ± 10.6
No. of adenomas removed	1	72%
	2	6%
	3	11%
	4	11%
Adenoma weight mean ± SD (mg)		683.7 ± 661
Total adenomas removed mean weight ± SD (mg)		1065 ± 812.5
Bone density ± SD (T-SCORE)		-3.0 ± 1.1
Kidney stones		16%
Symptoms (%)	Fatigue	79%
	Memory loss	37%
	Skeletal aches	53%
	Abdominal pain	21%
	Asymptomatic	10%
Surgical complications (%)	Transient hoarseness	5%
	Bleeding	0%
	Permanent complications	0%

**Table 2:** Mean scores before and after parathyroidectomy, improvement is score elevation.

Test	Before surgery (N=18)	1 m After surgery (N=18)	6 m after surgery (N=13)	p value
**RAVLT 8 mean score ± SD (0-15)**	7.0 ± 3.6	8.8 ± 3.3	8.5 ± 4.4	**0.002**
**ROCF 2 mean score ± SD (0-36)**	14.4 ± 7.4	18.7 ± 6.6	19.6 ± 9.5	**p<0.001**
**ROCF 3 mean score ± SD (0-36)**	14.5 ± 6.4	18.2 ± 7.1	19.4 ± 8.1	**p<0.001**
**FAB mean score ± SD (0-18)**	16.3 ± 1.7	16.8 ± 1.3	16.9 ± 1.7	0.082
**ACE TOTAL mean score ± SD (0-100)**	86.2 ± 8.4	88.3 ± 8.2	90.5 ± 7.0	**0.031**
**ACE Q5 mean score ± SD (0- 21)**	17.6 ± 3.2	18.1 ± 3.3	19.2 ± 2.2	**0.004**
**ACE Q5 DELAY mean score ± SD (0-7)**	5.2 ± 2.0	6.1 ± 1.4	6.2 ± 2.1	0.075

All p-values relate to improvement of scores between the three timelines.

Bold values show significant statistical p values.

Another improvement was seen on the patients’ performance of Trail Making Tests A and B wherein they both completed the tasks faster and more accurately (p value of <0.001 and =0.006, respectively). Thus, visual attention, mental flexibility, and executive functioning all improved after curative surgery. Interestingly, the performances of the patients in the Trail Making Test B were normalized since the standard deviation narrowed from one session to the next, as seen in **Table 3**.

**Table 3:** Mean scores before and after parathyroidectomy, improvement is score descent.

Test	Before surgery (N=18)	1m After surgery (N=18)	6 m after surgery (N=13)	p value
**Trail making Test A** **mean score (sec) ± SD**	56.3 ± 18.4	47.5 ± 16.7	41.9 ± 15.3	**p<0.001**
**Trail making Test B** **mean score (sec.) ± SD**	122.7 ± 66.0	95.6 ± 41.5	89.8 ± 36.1	**0.006**
**BDI mean score ± SD (0-63)**	5.9 ± 4.0	5.5 ± 5.3	5.2 ± 4.7	0.697

All p-values relate to improvement of scores between the three timelines.

Bold values show significant statistical p values.

Nevertheless, no change was seen in frontal lobe functions as measured *via* the FAB (mean score was ~16 – near normal - in all three sessions and with a non-significant p-value of 0.082). Although depression scores were improved after surgery (BDI), this did not reach statistical significance (p=0.697). It is important to note that the preoperative BDI score did not reach the lower limit of the depression range in any of the patients. (A score up to 10 is considered normal).

Regarding improvement rates for each test, 89% improved in the RCFT3; 78% improved in the RCFT 2, RAVLT 8 and Trail Making Test A; 67% improved in the Trail Making B test; 61% improved in the ACE 5 and BDI tests; 50% improved in the ACE 5 DELAY and FAB tests. All patients showed some improvement. The average number of tests improved was 6.9 ± 1.4 tests per patient.

No correlation was found between patients’ preoperative PTH nor calcium levels and their change in neurocognitive tests. Other variables that were tested for correlation with change in neurocognitive tests including age, total weight of adenomas removed, pre-operative serum 25OHD levels, failed to demonstrate significant correlation.

## Discussion

In this prospective study evaluating 18 PHPT patients for neurocognitive disorders before and 1 month after successful parathyroidectomy, a follow-up over 6 months in 13 of these patients was completed. The baseline impaired neurocognitive functions were: [1] the short- and long-term verbal memory with learning abilities assessed by RAVLT, [2] the short- and long-term visual memory, attention, planning, and working memory assessed by ROCF test, [3] visual attention, mental flexibility, and executive functioning evaluated by TMTs, and [4] complex neurocognitive functions (attention, memory, fluency, language, and visuospatial functions) assessed by ACE-III tests. This study demonstrated that successful parathyroidectomy resulted in a significant improvement in short- and long-term verbal memory and learning abilities (ACE5, RAVLT8). Moreover, a significant improvement in short- and long-term visual memory, attention, planning and working memory (ROCF2, ROCF3) was identified. Lastly, it demonstrated a significant improvement in visual attention, mental flexibility and executive function (TMT A, TMT B). The study did not identify an improvement in depressive symptoms (BDI) nor in frontal lobe-associated abilities (FAB). This is probably because all our patients had low baseline scores for depressive symptoms and near-normal baseline assessment of frontal capacities before surgery (mean FAB test results 16/18) making it difficult to identify any improvement of these functions. Neurocognitive impairment, mood and psychiatric disorders in PHPT patients were described as part of the general clinical symptoms in mid-twentieth century (usually retrospective) studies and were found in up to 65% of cases (23–26).

A review of the English-language literature concerning prospective, comprehensive evaluation of neurocognitive function before and after parathyroidectomy (5, 9–12, 27–37, 38), demonstrates that our results are in accordance with previous studies, but not with all, and add new data in terms of neurocognitive assessment before surgery, observed improvement after parathyroidectomy, and timing of improvement (**Table 4**). The tests used in prior studies were recurrent from one study to the other although not exactly similar, and could show a wide variability of both baseline range of impairment in verbal and visual memory, attention, complex neurocognitive functions, depression or anxiety symptoms, as well as post-surgery extent of improvement **Table 4**).

**Table 4:** Review of studies with neurocognitive evaluation of primary hyperparathyroidism patients at baseline and after parathyroidectomy.

	Number of patients, timing of NC evaluation AS.	Cognitive functions evaluated	Preoperatively findings	Post operative findings	Relation between NCS and PTH, Calcium, 25OHD
Cogan et al., 1978 (27).	19 included patients, but only 4 PHPT, 4 patients with secondary hyperparathyroidism, and 4 control patients were evaluated at B and at a mean of 3.6 months.	1-Raven’s Progressive Matrices 2-WAIS 3-Neurologic Index of Mental Impairment 4- VMI 5-Digit Symbol Test, 6-Finger Oscillation Test, 7- TMT 8-Memory for Designs Test, 9- PMS 10- Rotter’s Locus of Control Scale, 11-Watson’s Ego Strength Scale 12-Anxiety and Depression Scale 13-Barron’s Ego Strength Scale 14-MMPI	Weakness and fatigue in all groups of patients.	Improvement in weakness and fatigue only in patients with hyperparathyroidism. PHPT had no improvement in NC tests. Patients with secondary hyperparathyroidism had improvement in general cognitive function, nonverbal problem solving and visual-motor or visual spatial skills (Raven’s Progressive Matrices, VMI), sig- nificantly fewer errors on TMT, significantly better PMS.	NA
Numann et al., 1984 (28).	10 PHPT and 10 normocalcemic patients. Before and at 4 months.	1-short-term verbal memory and cognition (LM, WAIS). 2-Tests measuring conceptual tracking, short-term visual memory, graphic skills, and fine motor coordination	NA	Significant improvement in short-term memory and cognition. No change in any other evaluated NC function.	NA
Brown et al., 1987 (34).	34 PHPT, 19 PHPT were evaluated for neurocognitive avaluation with follow-up: 10 at 6 months AS and 9 at 6 months without surgery.	1-WAIS 2-TMT 3-STMDT 4- HFOT 5-MMPI (depression scale) 6-IQ	29% - normal evaluation 32% - affective disorders 39% cognitive abnormalities	No improvement in either PHPT after surgery or without surgery.	Correlation between calcium levels and motor speed, psychomotor speed, intelligence and short-term memory. No correlation with depression symptoms.
Goyal et al., 2001 (29).	14 PHPT, 12 MNG, 13 control patients addressed to gallbladder surgery, at 1, 3, 6 weeks and 3 and 6 months.	1-CPRS 2-Scale for Memory and Intelligence (for use in Hindi speaking population)	Impaired CPRS in PHPT.	Improvement in CPRS at 6 weeks: Sadness, lassitude, ache and pains, and fatigability.	No correlation
Roman et al. 2005 (9).	28 PHPT and 27 controls (neck surgery) at B. 41 patients evaluated at 4 weeks.	1-Depression (BDI) 2-Anxiety (STAI) 3- Verbal learning and memory (RAVLT) 4- Short term visuospatial memory (GMLT)	More depression and delays in visuospatial memory in PHPT, no difference between groups in anxiety and verbal memory.	Improvement in spatial learning and processing (RAVLT and GMLT).	Positive correlation between cognitive improvement and decrease in PTH
Chiang et al., 2005 (30).	20 PHPT and 20 matched orthopedic patients evaluated at B and a mean of 96 days for the former and 76 days for the latter patients.	1-Depression (BDI) 2-Anxiety (STAI) 3- Psychological symptoms (8SQ) 4-Perceptual set and suppression of habitual response (CW) 5-Perceptuo-motor coordination and attentional scanning (WAIS-R) 5-Verbal recent memory (RMMPT) 6- Visuo-spatial recent memory for designs (UST) 7-Memory, attention and concentration (Verbal and Visuo-spatial Yes/No Recognition Tests)	Only Verbal and Visuo-spatial Yes/No Recognition tests were significantly lower in PHPT but if no significance after statistical Bonferroni adjustment.	No significant improvement in PHPT in comparison to control group.	Association between decrease of calcium levels, Calcium and PTH levels at B with improvement in stress/psychologic symptoms (8QS) and mood (BDI, STAI). No significant correlation after Bonferroni adjustment.
Dotzenrath et al., 2006 (31)..	26 PHPT and 26 matched non toxic MNG evaluated at B. and 6 months.	1-Depression (HDRS) 2-visual scanning, numeric sequencing, and visuomotor speed (ZVT=TMT) 3-Intelligence (MWT) 4- Cognitive impairment (DEM Tect) 5- Cognitive impairment of visual memory (BVRT)	Before surgery, no PHPT complained about cognitive impairment, but 20/26 (79.3%) showed cognitive deterioration on evaluation at B.	Improvement in 50% of patients with depression or impaired NC evaluation. NO change in interlligence (MWT). No significant change in visual memory (BVRT).	No correlation between calcium or PTH levels and NC symptoms or improvement.
Mittendorf et al., 2007 (33).	55 PHPT, all evaluated for sleep disturbance and 47 evaluated for NCS at a median of 4 weeks.	1-Sleep disturbance(BSDI) 2- Executive functioning and Cognitive processing speed (CW)	Sleep disturbance in 44% of patients. 15/47 (32%) had impaired NC evaluation at B: impaired executive functioning and/or impaired cognitive processing speed.	Improvement of sleep disturbance in 46% of patients with baseline impairment. Significant improvement (80%) in executional functioning and/or in cognitive processing speed.	No correlation with calcium or PTH levels.
Perrier et al., 2009 (38).	18 PHPT (9 had parathyroidectomy, 9 were on clinical observation) evaluated at B, 6 weeks, and 6 months.	1-total sleep time and efficiency (wrist actigraphy) 2- Depression, memory, attention concentration, psychomotor speed and dexterity (BDI, WAIS, WAIS-R, VFT, GPT, TMT A and B, HVLTR, PASAT, STAI, CW)	Hypersomnolence and sleepiness.	Improvement in depression (BDI) and anxiety(STAI). Worsening hypersomnolence in the group on observation.	Correlation between change in sleep and change in PTH
Benge et al., 2009 (34).	167 PHPT patients had a preoperative neurocognitive evaluation, and 67 were evaluated at 1 month.	1-Depression (BDI) 2-Anxiety (STAI) 3-Common non specific complaints- health related quality of life (PAS) 4-Attention (WAIS) 5-Proceeding speed (PASAT) 6-Visual sequencing and processing speed (TMT A and WAIS-R) 7-Verbal learning and memory (HVLTR) 8-Executive function (TMT B) 9- Verbal fluency (VFT) 10-Timed inhibition of competing stimuli/processing speed measures (CW) 11-Fine motor speed/dexterity (GPT)	35.1% of PHPT patients had preoperative CSI of at least one neurocognitive evaluation. 30.6% had CS depression and 17.1% CS anxiety	Improvement in fine motricity (GPT), information processing speed (PASAT, TMTA), Depression improved in 28.3% of patients, only 6.2% remained with anxiety. Mixed results regarding overall NCS: improvement in 37.5% of patients with initially CSI. 18% of initially non-CSI patients had CSI after surgery (52.3% memory, 19%VFT, 14.1% PASAT, 9.5% TMT, 4.5% CW)	No significant correlation
Walker et al. 2009 (5).	39 PM women with PHPT and 89 PM controls without PHPT. Evaluation at B. and at 6 months.	1-IQ (NAART), 2-Depression (BDI), 3-Anxiety (STAI), 4-Memory for contextually related material (LM) 5-Word List memory (SRT), 6-Visual Memory (RVDLT) 7-Non verbal Abstraction (BCT) 8-Visual concentration/attention (RTD and WAIS-R) 9- Auditory attention (WAIS)	More depression and anxiety, impairment in verbal memory (LM and SRT) and non verbal abstraction (BCT) in PHPT. No difference in visual memory (RVDLT), visual concentration and attention (RTD and WAIS-R), auditory attention (WAIS)	Improvement in depression, verbal memory (LM), visual memory (RVDLT), non verbal abstraction (BCT). Improvement in visual concentration and attention in PHPT patients but not in controls.	No relation between PTH, calcium, nor 25OHD levels at preoperative levels and neurocognitive baseline impairment, and no relation between change in levels of PTH, calcium or 25OHD and neurocognitive changes after the surgery.
Roman et al., 2011 (10).	212 PHPT. 159 patients were evaluated at 1 month, 117 at 3 months, and 102 at 6 months.	1-Depression (BDI) 2-Anxiety (STAI) 3-Global psychological distress (BSI-18) 4-Verbal and visual learning (RAVLT, GMLT).	Mean scores showed PHPT had mild depression, increased anxiety trait and global stress. Verbal and visual learning were within normal range at B.	Improvement in depression scores and global stress (BDI and BSI-18), in STAI- Anxiety State but not in STAI -Anxiety Trait. Improvement in verbal and visual learning AS (RAVLT, GMLT).	Decrease in PTH wasmodestly associated with improvement in anxiety and cognitive function
Babinska et al., 2012 (11).	35 PHPT patients and 35 matched controls addressed to another surgery. Evaluation at B. and 12 and 18 months.	1-Depression (BDI) 2-Visual Perception, visual memory and visual constructive abilities (BVRT) 3- Operative memory WCTS 4- Visual memory (DCS) 5- Verbal learning and Memory (RAVLT) 6-Visuospatial memory and attention (TMT) 7- Verbal fluency, semantic and verbal memory (VFT)	Higher depression score (BDI) but within normal range in PHPT patients. Significant concentration and visual memory impairment (BVRT and DCS), decreased non verbal learning process, direct memory and visuospatial memory/attention focus (TMT A) as well as verbal fluency (VFT) in PHPT patients.	Improvement in depression score (BDI) in PHPT patients, in direct memory, verbal memory (RAVLT), in visual memory (BVRT), in visual attention (TMT A), but no straight improvement in TMT B.	No correlation between calcium or PTH preoperative leves and neurocognitive impairment,
Trombetti et al., 2016 (35).	332 PHPT (multicenter), 185 had B. NC evaluation, 112 were on follow-up yearly. 44 patients were re-evaluated after successful parathyroidectomy 3-6 months AS. 17 patients were on follow-up without surgery.	1-Depression and anxiety (HADS) 2-Cognitive testing (MMSE and CDT)	25% of all PHPT displayed a pathological MMSE. 47% had abnormal CDT. 33% had pathological HADS anxiety and 20% HADS depression.	Parathyroidectomy significantly improved scores in MMSE, HADS anxiety and depression but not in the CDT. No change in PHPT without surgery.	Negative correlation between serum PTH and MMSE and CDT, no correlation with calcium or 25OHD.
Shah et al., 2018 (12).	50 PHPT patients evaluated at B. and 35 patients were evaluated at 1 week and 16 at 3 months.	1-Depression and anxiety (HADS) 2-Verbal Learning and memory (RAVLT) 3-Attention, speed and mental flexibility (TMT A and B). 4-Verbal fluency (VFT) 5-Attention and working memory (WAIS) 6- Positive and negative emotions (PANAS) 7-Sleep disturbance (ISI)	Impaired immediate memory, delayed recall, delayed recognition. Borderline anxiety and depression in 74% of patients, overt anxiety and depression in 26%. Moderate sleep disturbance in 56%.	Early improvement (at 1 week) in working memory, delayed memory, attention, mood and sleep (RAVLT, TMT A, HADS, ISI) but no change in TMT B, no significant improvement in WAIS and in VFT. At 3 months, sustained improvement in NCS and also verbal fluency (VFT).	No correlation between preoperative calcium and PTH levels and symptoms.
Liu et al., 2020 (36).	29 PHPT, 7 normocalcemic PHPT and 7 MNG, evaluated at B. 23/29 PHPT and 5/7 MNG evaluated at 6 months.	1-Depression (CESD) 2- Visual attention, mental flexibility, processing speed (Letter cancellation, TMT A and B). 3- Verbal memory, fluency and word retrieval (HVLT, CW, BNT,RPST) 4- Visuo-spatial perception, constructional ability, visual memory (ROCFT). 5- Working memory and mental manipulation (WAIS, WAIS-R). 6- Motor speed and coordination (GPT). 7- Spatial neglect (LBT).	No detected NC impairment and no significant difference in PHPT in comparison to other patients (to note, MNG had lower performance in CW).	Significant improvement in depression symptoms in PHPT (43%). Mild improvement in coordination (GPT) but worsening visual recognition (HVLT).	Relation between low 25OHD and impaired WAIS. Higher Calcium levels correlated with depressive symptoms. PTH levels associated with lower performance in visual attention (TMT A), verbal memory (RPST) and motor coordination (GPT).

AS, After surgery; B, Baseline; BCT, Booklet Category Test, Victoria Revision; BDI (2^nd^ edition) – Beck Depression Inventory; BNT, Boston Naming Test; BVRT, Benton Visual Retention Test- Method A; BSDI, Brief Sleep Disturbance Inventory; BSI-18, Brief Symptom Inventory–18; CDT, Clock Drawing Test; CESD, Center for Epidemiological Studies Depression Scale; CPRS, Comprehensive Psychopathological Rating Scale; CS, clinically significant; CSI, clinically significant impairment; CW, Stroop Color-Word trial/Stroop Word Subtest and Stroop Color Subtest; DCS- Memory Verbal Learning Test by F.Hillers; GMLT, Groton Maze Learning Test; HADS, Hospital Anxiety and Depression Scale; HDRS, Hamilton Depression Rating Scale; GFOT, Hamelstead’s finer oscillation test; HVLTR, Hopkins Verbal Learning Test-Revised; IQ, Intellectual Quotient; ISI, Insomnia Severity Index; GPT, Grooved Pegboard test; LBT, Line Bisection test; LM, Wechsler Memory Scale Logical Memory Test, Russel Revision; MMPI, Minnesota Multiphasic Personality Inventory; MMSE, mini mental state evaluation; MNG, multinodular goiter patients; MWT, multiple word test (German population); NA, not available; NAART, North American Adult Reading Test; NCS, neurocognitive symptoms; PANAS, Positive Affect Negative Affect Schedule; PAS, Parathyroidectomy Assessment of Symptoms Scale; PASAT, Paced Auditory Serial Addition Test; PM, post menopausal; PMS, Profile of Mood States; RAVLT, Rey Auditory Verbal Learning Test; RMMPT, Royal Melbourne Memory for Prose Test; ROCFT, Rey-Osterreith Complex Figure Test; RPST, Repetition of Phrases and Sentences test); RVDLT, Rey Visual Design Learning Test; RTD, Rosen Target Dectection Test; 8SQ, Eight StateQuestionnaire; SRT , Buschke Selective Reminding Test; STAI- State-Trait Anxiety Inventory; STMDT, short term memory distractor test; TMT, Trail Making Test; UST, Unusual Shapes Test; VMI, Visual Motor Items; VFT, Verbal Fluency Test (Controlled Oral Word Association Test); WAD, Wechsler Adult Intelligence Scale Digit Span Subtest; WAIS-R, Wechsler Adult Intelligence Scale-Revised Digit Symbol Subset; WCST- Wiscinsin Card Sorting Test; ZVT, Zahlenverbindungstest.

Some subtle differences in retrieval verbal memory versus identification verbal memory capacity or phonetic and semantic verbal flux warrant further investigation for better characterization in patients with hyperparathyroidism. Of note, our study is the first to show a significant improvement in the TMT B related to amelioration of visual attention dysfunction, whereas Babinska et al. (11) as well as Shah et al. (12) failed to demonstrate such improvement. Moreover, difference in the timing of improvement from one study to the other can be observed, which may be due to the different recruited populations and the heterogeneous design of the studies.

The recently published European expert consensus on practical management of specific aspects of parathyroid disorders as part of the recommendations of the European Society of Endocrinology Program of Parathyroid Disorders (PARAT2021) did not assess at all the issue of neurocognitive impairment in PHPT patients (14). However, the recently-updated Fifth International Workshop on the Evaluation and Management of Primary Hyperparathyroidism recommended avoiding performing routine neurocognitive evaluation and to avoid using baseline neurocognitive impairment to decide on surgery (8). The main argument was about evaluation of quality of life (QoL) in PHPT patients (39): non-specific and subjective QoL questionnaires as well as short form 36-item (SF-36) have been used in the past and showed decreased QoL in PHPT patients, with improvement after successful parathyroidectomy (40–42); however, a recent well-designed Scandinavian prospective study showed no significant difference in QoL in PHPT patients during 10 years of follow-up after parathyroidectomy in comparison with those without surgery (43). Similarly, the 2019 first European workshop on parathyroid only raised the ambiguous results concerning potential benefit of parathyroidectomy on QoL (44). However, the debate is not closed as a recent large review showed that despite heterogenous designs in the 31 included studies, PHPT was associated with reduced QoL and several parameters (vitality, mental health, mood swings) improved significantly after parathyroidectomy when appropriately assessed either by the Parathyroidectomy Assessment of Symptoms Scale (PAS) or the 36-item Short Form Survey (SF-36) (45). Moreover these positions were actually based on a very limited aspect of impaired cognition in PHPT patients, and did not consider the studies which described extended neurocognitive assessment in PHPT patients.

Thus, we believe that performing a neurocognitive assessment beyond simple evaluation of QoL, but rather focusing on verbal, visual, visuospatial, attention, and executive functioning assessment, as observed in our study and many others, may reveal the potential benefit of parathyroid surgery in those patients for whom there is no clear-cut decision based on the classical surgical criteria as updated by the Fifth International Workshop on Parathyroidectomy. Indeed, the observed improvement following parathyroidectomy may have a significant impact on the day-to-day life of patients with PHPT, for example due to improved capacity to remember a list of words or to plan and execute simple tasks.

Finally, despite some studies demonstrating a correlation between baseline PTH levels and impaired neurocognitive assessment, or extent of decrease in PTH levels after parathyroidectomy and neurocognitive improvement, many other studies, including the present one, found no correlation between PTH, calcium or 25OHD levels and pre-operative neurocognitive function deterioration or post-surgery improvement (**Table 4**). However, the correlation between 25OHD levels and cognitive impairment was rarely evaluated.

A systematic review included 27 studies of low to moderate quality with evaluation of relation between PTH and dementia or cognitive impairment, found mixed results and potential improvement in memory after parathyroidectomy (46); A more recent systematic review (47) identified two studies that demonstrated potential correlation between elevated PTH levels and alteration in the Mini Mental State Examination. However, the quality of evidence was low overall, with heterogenous results for other neurocognitive evaluations. Another study (which did not focus on patients with primary hyperparathyroidism) did not show any relation between elevated PTH levels and progressive cognitive decline over 20 years as evaluated by three tests (Delayed Word Recall, the Digit Symbol Substitution, and the Word Fluency tests) and a compound Z score (48). On the other hand, analyses of calcium and monoamine in the cerebral spine fluid were found to correlate with neurocognitive impairment and improvement after surgery (49, 50); data from a recent review did not demonstrate that changes in CSF could be related to neurocognitive symptoms (51).

These observations underscore the fact that we are still far from understanding the pathophysiology underlying neurocognitive symptoms in PHPT patients. However there is a physiologic basis to support the association between PHPT and neurocognitive symptoms, as co-distribution in specific regions of the brain of parathyroid hormone 2 receptor (PTH2R) and an endogenous peptide (tuberoinfundibular peptide of 39 residues, able to locally activate PTH2R) suggested the existence of a neuromodulatory system in specific regions of the brain (52) which can be involved in auditory and limbic functions as well as stress regulation or anxiety levels. Several studies (32, 36, 38, 53, 54) examined dysfunction in specific cerebral zones using functional resonance magnetic imaging, alteration in vertebral vascular flow using transcranial doppler (36) or single photon emission computed tomography (55), or perioperative changes in cortical excitability with transcranial magnetic stimulation (56) in patients with PHPT. However, these studies have not yet reach a systematic and homogeneous conclusion (16).

The findings of our study are concordant with those of previously studies (**Table 4**), and show that specific subtle neurocognitive function are repeatedly found to be altered in PHPT patients and may improve after successful parathyroidectomy.

Our study has several limitations. First, it includes a relatively small sample of patients, which limits the extension of our results to larger cohorts. Unfortunately, the coronavirus pandemic prevented us from continuing to perform in-home interviews and testing. Second, the study design did not include a control group of patients who did not undergo parathyroidectomy. Yet, each patient is compared to his/her own results before surgery, and the intention of this study was to focus on the effect of successful surgery. Third, patients’ pre-surgery neurocognitive assessments demonstrated only mild impairment in most of the tests, and were normal in some, which of course limits the possibility to identify any improvement after surgery.

Despite these important limitations, our study did demonstrate that in patients with PHPT, parathyroidectomy leads to rapid (within one month) improvement in several neurocognitive functions such as short- and long-term verbal memory, short- and long-term visual memory, visual attention, and concentration. Other functions such as complex cognitive functions and global management capacity improve later (at 6 months). This study suggests that parathyroidectomy may positively impact daily neurocognitive functions of PHPT patients, with important improvement in several day-to-day activities. A practical approach to identify PHPT patients without straight indication for parathyroidectomy but who could benefit from surgery, would be to evaluate QoL and to screen neurocognitive impairment by asking during a consultation a simple question to the patient as: “Do you feel you have any problems with your thinking or memory?” and separately to family members: “Do you feel he/she has any problems with his/her thinking or memory?”; according to the answers, a decision can be taken to address or not the patient for further specific neurocognitive evaluation as described in our study.

As recommended in the research agenda published by the Fifth International Workshop on Primary Hyperparathyroidism (13), larger prospective randomized controlled trials are essential to determine the optimal neurocognitive evaluation battery tests before parathyroidectomy and to evaluate the benefit of the latter. A systematic approach and further obtention of appropriate data will determine if altered neurocognitive function may be considered a potential new surgery criterion for patients with primary hyperparathyroidism who do not meet the strict consensual criteria for parathyroidectomy.

## Data availability statement

The original contributions presented in the study are included in the article/supplementary material. Further inquiries can be directed to the corresponding author.

## Ethics statement

The studies involving human participants were reviewed and approved by Hadassah Medical Center Ethics Committee. The patients/participants provided their written informed consent to participate in this study.

## Author contributions

AS - Design, writing and reviewing. NT - Retrieving data, analysis of data, writing. HM - Design, retrieving data, writing, reviewing JN - Design, reviewing. All authors contributed to the article and approved the submitted version.
